# Zika Virus Infection Preferentially Counterbalances Human Peripheral Monocyte and/or NK Cell Activity

**DOI:** 10.1128/mSphereDirect.00120-18

**Published:** 2018-03-28

**Authors:** Fok-Moon Lum, David Lee, Tze-Kwang Chua, Jeslin J. L. Tan, Cheryl Y. P. Lee, Xuan Liu, Yongxiang Fang, Bernett Lee, Wearn-Xin Yee, Natasha Y. Rickett, Po-Ying Chia, Vanessa Lim, Yee-Sin Leo, David A. Matthews, Julian A. Hiscox, Lisa F. P. Ng

**Affiliations:** a Singapore Immunology Network, Agency for Science, Technology and Research (A*STAR), Singapore, Singapore; b School of Cellular and Molecular Medicine, University of Bristol, Bristol, United Kingdom; c NUS Graduate School for Integrative Sciences and Engineering, National University of Singapore, Singapore, Singapore; d Centre for Genomic Research, Institute of Integrative Biology, University of Liverpool, Liverpool, United Kingdom; e National Institute of Health Research, Health Protection Research Unit in Emerging and Zoonotic Infections, University of Liverpool, Liverpool, United Kingdom; f Institute of Infection and Global Health, University of Liverpool, Liverpool, United Kingdom; g Communicable Diseases Centre, Institute of Infectious Diseases and Epidemiology, Tan Tock Seng Hospital, Singapore, Singapore; h Lee Kong Chian School of Medicine, Nanyang Technological University, Singapore, Singapore; i Saw Swee Hock School of Public Health, National University of Singapore, Singapore, Singapore; j Department of Biochemistry, Yong Loo Lin School of Medicine, National University of Singapore, Singapore, Singapore; Icahn School of Medicine at Mount Sinai; University of Pittsburgh; University of North Carolina at Chapel Hill

**Keywords:** NK cells, RNA-seq, Zika virus, immune response, monocytes, transcriptomes

## Abstract

Zika virus (ZIKV) has reemerged in the population and caused unprecedented global outbreaks. Here, the transcriptomic consequences of ZIKV infection were studied systematically first in human peripheral blood CD14^+^ monocytes and monocyte-derived macrophages with high-density RNA sequencing. Analyses of the ZIKV genome revealed that the virus underwent genetic diversification, and differential mRNA abundance was found in host cells during infection. Notably, there was a significant change in the cellular response, with cross talk between monocytes and natural killer (NK) cells as one of the highly identified pathways. Immunophenotyping of peripheral blood from ZIKV-infected patients further confirmed the activation of NK cells during acute infection. ZIKV infection in peripheral blood cells isolated from healthy donors led to the induction of gamma interferon (IFN-γ) and CD107a—two key markers of NK cell function. Depletion of CD14^+^ monocytes from peripheral blood resulted in a reduction of these markers and reduced priming of NK cells during infection. This was complemented by the immunoproteomic changes observed. Mechanistically, ZIKV infection preferentially counterbalances monocyte and/or NK cell activity, with implications for targeted cytokine immunotherapies.

**IMPORTANCE** ZIKV reemerged in recent years, causing outbreaks in many parts of the world. Alarmingly, ZIKV infection has been associated with neurological complications such as Guillain-Barré syndrome (GBS) in adults and congenital fetal growth-associated anomalies in newborns. Host peripheral immune cells are one of the first to interact with the virus upon successful transmission from an infected female *Aedes* mosquito. However, little is known about the role of these immune cells during infection. In this work, the immune responses of monocytes, known target cells of ZIKV infection, were investigated by high-density transcriptomics. The analysis saw a robust immune response being elicited. Importantly, it also divulged that monocytes prime NK cell activities during virus infection. Removal of monocytes during the infection changed the immune milieu, which in turn reduced NK cell stimulation. This study provides valuable insights into the pathobiology of the virus and allows for the possibility of designing novel targeted therapeutics.

## Abstract (precis)

ZIKV reemerged in recent years, causing outbreaks in many parts of the world. Alarmingly, ZIKV infection has been associated with neurological complications such as Guillain-Barré syndrome (GBS) in adults and congenital fetal growth-associated anomalies in newborns. Host peripheral immune cells are one of the first to interact with the virus upon successful transmission from an infected female *Aedes* mosquito. However, little is known about the role of these immune cells during infection. In this work, the immune responses of monocytes, known target cells of ZIKV infection, were investigated by high-density transcriptomics. The analysis saw a robust immune response being elicited. Importantly, it also divulged that monocytes prime NK cell activities during virus infection. Removal of monocytes during the infection changed the immune milieu, which in turn reduced NK cell stimulation. This study provides valuable insights into the pathobiology of the virus and allows for the possibility of designing novel targeted therapeutics.

## INTRODUCTION

Zika virus (ZIKV) gained global attention in 2015 to 2016 when the virus suddenly reemerged in the human population and caused major viral outbreaks across the world with a large disease burden (1). Although ZIKV has been causing sporadic outbreaks since it was first reported in Uganda >60 years ago (2), very little is known about the biology of the virus and the host response to infection. ZIKV is an arthropod-borne flavivirus that causes Zika fever—a disease that for the majority of patients has few or no symptoms (3). However, in severe cases, ZIKV infection may be responsible for neurological complications such as Guillain-Barré syndrome (GBS) in adults (4) and congenital fetal growth-associated anomalies in newborns (5). The host response to ZIKV infection may be one of the main drivers of the different disease phenotypes.

Recent studies have established that ZIKV can infect peripheral blood monocytes (6–9). However, despite ongoing intensive investigative efforts to understand ZIKV-related neuropathogenesis, knowledge regarding the mechanisms of ZIKV infection in peripheral immune cells is lacking. Given that ZIKV is transmitted into the dermis via the bite from a virus-infected mosquito, monocytes would be one of the first immune cells in the blood to interact with the virus when it reaches the circulatory system. Therefore, the interplay between ZIKV and monocytes will be crucial in determining the outcome of infection (10).

This study focused on characterizing the primary *ex vivo* response of human donor blood monocytes and monocyte-derived macrophages (MDMs) to ZIKV infection. Systematically, RNA sequencing (RNA-seq) was first used to identify and quantify the abundance of host mRNA and characterize viral RNA. This information was subsequently used to map the host response to ZIKV infection in the two different *ex vivo* cell types. These data also provided insights into the potential adaptation of the virus during viral replication in these cells. Immunophenotyping of peripheral blood cells isolated from patients infected with ZIKV independently was executed to validate the predictions obtained from the differential gene expression analysis. Depletion of CD14^+^ monocytes in peripheral blood was then performed *ex vivo* to functionally understand the cross talk between monocytes and priming of NK cells during ZIKV infection. Last, a multiplex assay was carried out to further understand host cell immunoproteomic changes during ZIKV infection. This global analysis of the host immune response provides a novel understanding of the pathobiology of the virus, leading to the possibility of targeted therapeutic interventions in severe cases.

## RESULTS

### ZIKV targets human peripheral blood monocytes and macrophages.

**FIG 1:**
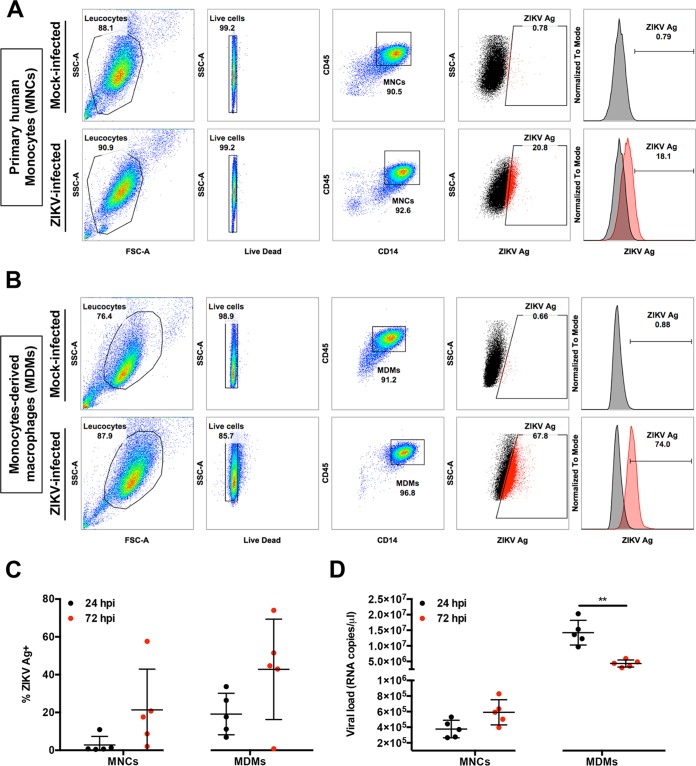
Primary human MNCs and MDMs are targets of ZIKV infection. Isolated human primary MNCs and MDMs (2 × 10^6^ cells each) were infected with ZIKV at an MOI of 10 and harvested at 24 and 72 hpi. (A and B) Flow cytometry gating on monocytes (MNCs) (A) and MDMs (B). Gating for positive infection was set using the mock-infected samples. For the dot plots, cells positive for ZIKV antigen (Ag) are shown in red. For the histogram, ZIKV-infected samples (red) were overlaid on mock-infected samples (black). Gating of live cells was performed on single cells. SSC, side scatter; FSC, forward scatter. (C and D) Compiled results for infection (ZIKV Ag) (C) and viral load detected in MNCs and MDMs obtained from five healthy donors (D). All data are presented as means ± standard deviations. **, *P* < 0.05, by Mann-Whitney *U* test, two-tailed. Viral load data were not statistically significant between 24 and 72 hpi in MNCs by Mann-Whitney *U* test, two-tailed.

CD14^+^ monocytes have been reported to be the main targets of ZIKV during infection (6–9). In this study, human primary CD14^+^ monocytes were first isolated from fresh peripheral blood mononuclear cells (PBMCs) to enrich this cell type to >90% of the total cell population (Fig. 1A). In addition, isolated monocytes from the same donors were differentiated into monocyte-derived macrophages (MDMs) over 5 days (Fig. 1B). Purified cells were then infected *ex vivo* with ZIKV, and their permissiveness to ZIKV infection and growth was determined at 24 and 72 h postinfection (hpi) (Fig. 1A). The 24-hpi time point was chosen to represent the acute infection phase, and the 72-hpi time point was chosen to represent a stage by which a substantial host-virus interaction would have taken place (11). Data obtained showed that ZIKV infection of MDMs was more significant than infection of monocytes in all five donors (~40% compared to ~20% at 72 hpi, respectively) (Fig. 1C). A decrease in viral load was observed in the virus-infected MDMs between the two time points, whereas the viral load remained consistent in infected monocytes over time (Fig. 1D). RNA-seq was then performed to assess global mRNA abundance in these cells.

### Genome variation in ZIKV during infection of the peripheral blood.

In order to compare the amounts of virus between the different cell types and determine whether ZIKV underwent genetic diversification during infection, viral sequence reads (obtained from the RNA-seq) were mapped and compared to that of the progenitor virus stock (PF/ZIKV/HPF/2013). These data indicated that for MDMs, 4.53% and 0.43% of total sequence reads (~10 to 140 million reads) mapped to the ZIKV genome at 24 hpi and 72 hpi, respectively, while 24% and 0.8% of sequence reads (~50 to 120 million reads) generated from monocytes mapped to the ZIKV genome at 24 hpi and 72 hpi, respectively.

**FIG 2:**
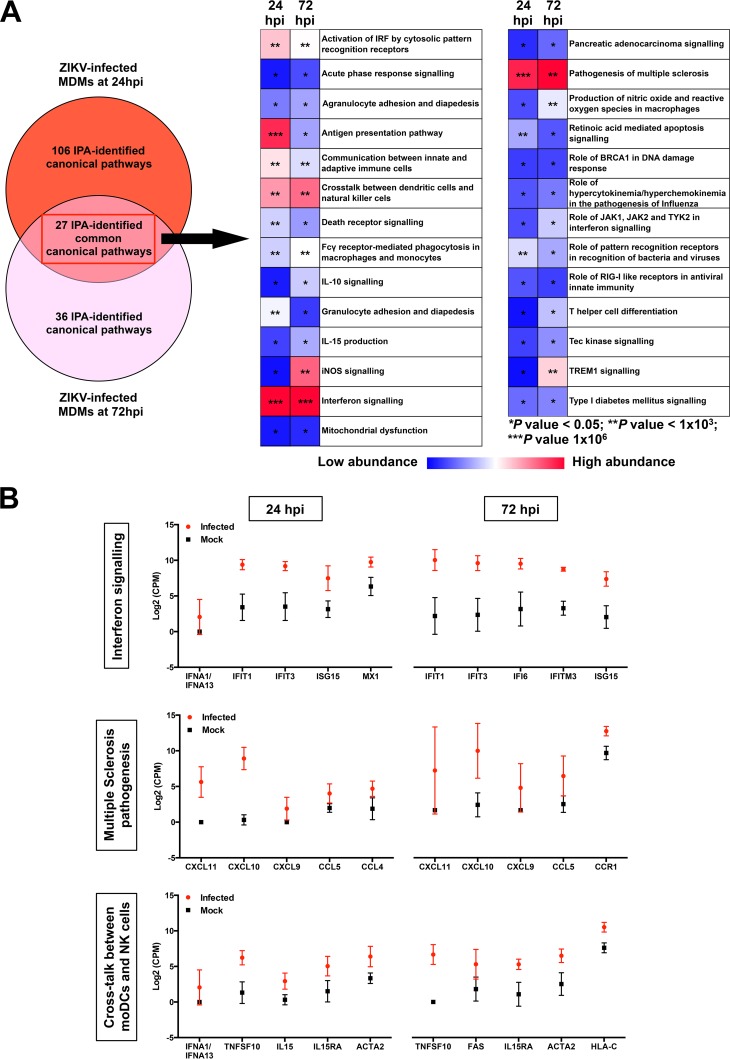
Transcriptomic profiling of host cells during ZIKV infection. Primary human MNCs and MDMs (2 × 10^6^ cells per infection) were infected with ZIKV at an MOI of 10, harvested at 24 and 72 hpi for transcriptomic analysis by RNA-seq, and then compared to mock-infected controls. (A) Venn diagram illustrating the proportion of upregulated signaling pathways identified by IPA in ZIKV-infected MDMs. Upregulation intensities of the 27 common canonical pathways are shown in a heat map. Asterisks within the boxes represent the calculated *P* values associated with each identified pathway compared to the mock-infected samples. (B) The five most upregulated genes within the top three signaling pathways (interferon pathway, multiple sclerosis pathway, and cross talk between moDCs and NK cells) at 24 and 72 hpi are shown. Data presented were obtained from a total of five donors.

Due to the inherent error-prone nature of viral RNA replication, nucleotide variants may become established in the viral genome during ZIKV infection in different cell types. To investigate this hypothesis, consensus genome information for each sample and the frequency of minor variants at each nucleotide position in the progenitor stock were determined and compared to the genome of virus present in the infected samples utilizing previously developed workflows (12, 13). The ZIKV consensus genome sequence derived from the progenitor stock was 10,570 nucleotides in length and contained minor variants (as a measure of quasispecies) spread throughout the genome (see Fig. S1A in the supplemental material). Of the 11 valid consensus sequences derived from the virus-infected samples, the virus recovered in cells from five donors (D1 to D5) had the same consensus sequence as the input stock (PF/ZIKV/HPF/2013). However, some donor samples contained viral genomes that had additional nucleotide differences at six different positions (Table S1). These nucleotide differences (Table S1) were visualized as a maximum likelihood phylogenetic tree, where the input stock was used as the reference sample (Fig. S1B). There were only eight high-frequency transition mutations to choose from (log_10_8 = 0.9 [Fig. 2A]), increasing the likelihood of these changes appearing several times. Of these eight transition mutations, six appeared as major variants and thus changed the overall consensus sequence. The nucleotide positions of these six transition mutations (Table S2) indicated that all the changes in the consensus sequence were already present at relatively high frequency as minor variants in the input stock and were subsequently amplified during viral replication. Changes at nucleotide positions 2815 and 4211 were the most common, being found in ~35% of reads mapping to the virus genome. Had these changes been found in ≥50% of reads, they would have been classified as major variants and thus changed the consensus sequence (Table S2).

10.1128/mSphereDirect.00120-18.1 FIG S1 Phylogenetic analyses based on sample consensus sequences. (A) Frequency of ZIKV minor variants (transitions and transversions) recovered from infected human primary MNCs and MDMs isolated from five donors. Bin −3 is where ≤1/1,000 reads show a specific change at an individual nucleotide position. Bin −2 is >1/1,000 and ≤1/100 reads showing a difference. Bin −1 is >1/100 and ≤1/10 reads, and bin >−1 is >1/10 reads showing a change up to a logical limit of just under 1/2. (B) Phylogenetic tree generated from the alignment of consensus sequences of ZIKV RNA recovered from the same samples as described for panel A. All samples included in the tree had a mean sequence coverage of >10 at each nucleotide position. PF/ZIKV/HPF/2013 is the virus strain used for infection and denoted as the reference sample in this analysis. Download FIG S1, PDF file, 0.1 MB. Copyright © 2018 Lum et al. 2018 Lum et al. This content is distributed under the terms of the Creative Commons Attribution 4.0 International license.

10.1128/mSphereDirect.00120-18.6 TABLE S1 Summary of nucleotide differences at specific genome positions. Download TABLE S1, PDF file, 0.1 MB. Copyright © 2018 Lum et al. 2018 Lum et al. This content is distributed under the terms of the Creative Commons Attribution 4.0 International license.

10.1128/mSphereDirect.00120-18.7 TABLE S2 Selected nucleotide positions from the minor variant file of the inoculum. Download TABLE S2, PDF file, 0.1 MB. Copyright © 2018 Lum et al. 2018 Lum et al. This content is distributed under the terms of the Creative Commons Attribution 4.0 International license.

### Transcriptomic profiling reveals key cellular responses to ZIKV infection.

RNA-seq was used to identify and quantify global mRNA abundance in ZIKV-infected peripheral monocytes and MDMs at 24 and 72 hpi. For monocytes, mock- and ZIKV-infected cells at both 24 and 72 hpi exhibited minimal changes in host transcript abundance. For MDMs, the abundances of transcripts that mapped to 1,736 and 545 genes at 24 and 72 hpi, respectively, were significantly different (false discovery rate [FDR] of <0.05) between the mock- and ZIKV-infected samples.

Ingenuity pathway analysis (IPA) was used to interrogate and group the differentially expressed genes into functional pathways (Fig. 2A). Of all the canonical pathways identified, 27 were common in ZIKV-infected MDMs at 24 and 72 hpi (Fig. 2A). This analysis found that genes associated with the interferon response were significantly upregulated at both time points. In addition, signaling pathways involved in the pathogenesis of multiple sclerosis and key pathways involved in monocyte-derived dendritic cell (moDC) and NK cell processes were also shared between the two time points (Fig. 2A). Overall, the top three common pathways activated in MDMs were interferon signaling, multiple sclerosis pathogenic pathways, and cross talk pathways between moDCs and NK cells (Fig. 2A). The specific genes with the most abundant transcripts within these three pathways were analyzed and, compared to the mock-infected controls, were all increased in abundance after ZIKV infection (Fig. 2B).

### Virus-infected MDMs exhibit reduced cellular responsiveness.

**FIG 3:**
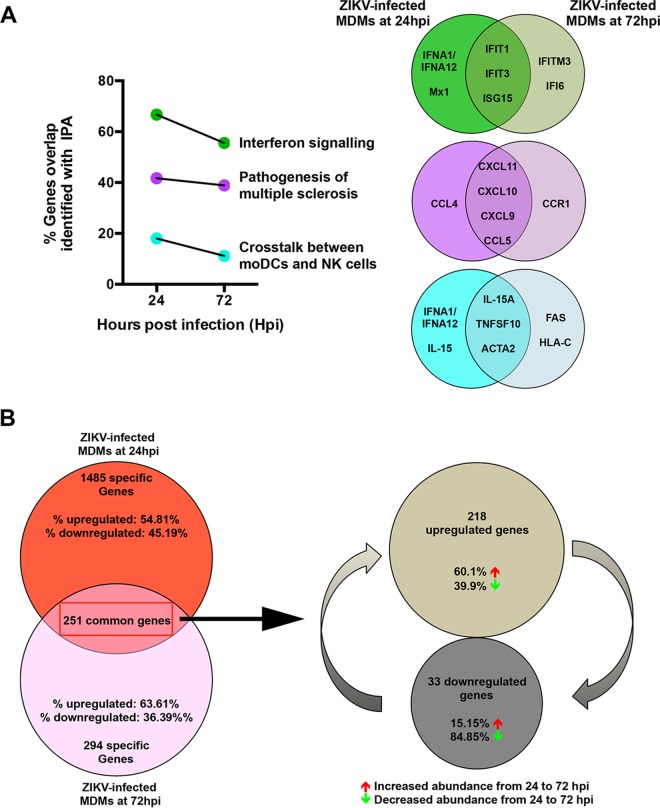
Transition of the host cellular response over the course of ZIKV infection. The host cellular response was analyzed and investigated by RNA sequencing, and significant transcriptomic differences were identified. (A) Transitional analysis (percentage of genes overlapping) of the top three common canonical signaling pathways was performed using IPA of infected MDMs. Venn diagrams indicate the top five common and time-point-specific genes associated with each canonical pathway. (B) Proportion of common and differentially expressed genes within ZIKV-infected MDMs at 24 and 72 hpi. Data presented were obtained from a total of five donors.

Transcriptomic profiles of various ZIKV-infected MDMs were compared to evaluate the transition of the cellular host response over the course of ZIKV infection. The percent overlap of the identified transcripts between ZIKV-infected MDMs was assessed at 24 hpi and 72 hpi within the three targeted pathways described above (Fig. 3). Interestingly, the percentage of overlapping transcripts identified at 72 hpi was lower for all three pathways, which may reveal a reduced activation status of these pathways at this stage of the infection. The identification of different transcripts associated with 72 hpi may indicate the different signaling cascades present or the activation status of these cells (Fig. 3A). Global assessment of all identified transcripts revealed that transcripts mapping to 251 genes were in fact present in virus-infected MDMs at both time points. Transcripts that mapped to 1,485 genes were specific to 24 hpi, of which 54.81% exhibited increased abundance compared to the mock controls. By comparison, transcripts that mapped to 294 genes were unique to 72 hpi, with 63.36% of them having increased mRNA abundance compared to the mock controls (Fig. 3B). Within the 251 common genes, transcripts mapping to 218 genes had an increased fold change value compared to the mock-infected controls, indicating that these transcripts were increased in abundance in all ZIKV-infected MDMs. Further inquiry of these transcripts revealed that 60.1% of them were increased in abundance at 72 hpi compared to 24 hpi. Likewise, of the remaining transcripts that mapped to 33 genes and showed decreased abundance, 84.85% were further reduced at 72 hpi.

### NK cells are activated in ZIKV-infected patients.

**FIG 4:**
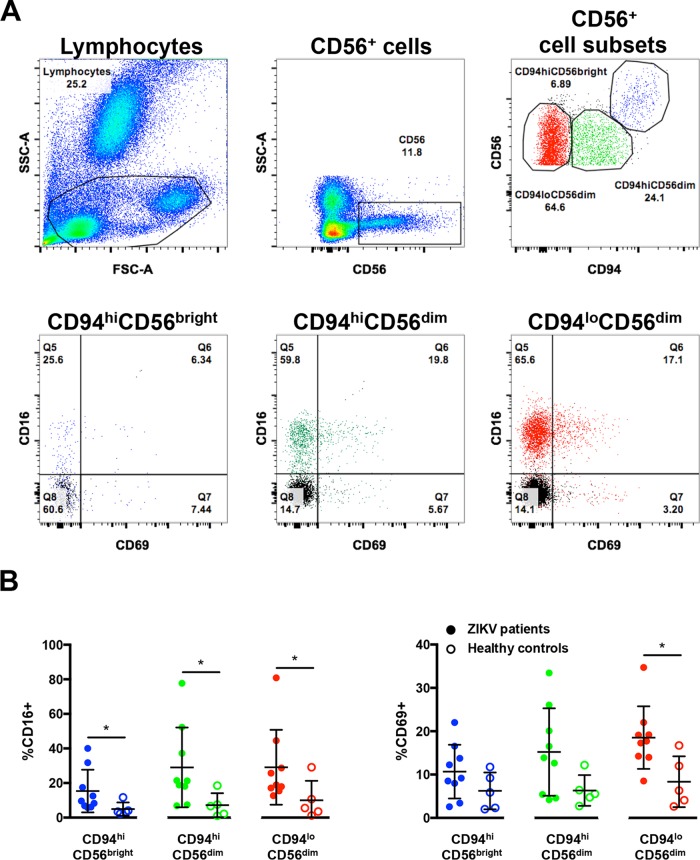
Activation of CD56^+^ cells in patients infected with ZIKV. (A) Gating strategy of CD56^+^ cell subsets and their expression of CD16 and CD69. Lymphocytes were first gated to exclude the neutrophils. Subsequently, CD56^+^ cells were identified and further gated into three populations based on the expression of surface marker CD94: CD94^hi^ CD56^bright^ (blue), CD94^hi^ CD56^dim^ (green), and CD94^lo^ CD56^dim^ (red). The data presented correspond to a representative patient infected with ZIKV. Cells from a healthy control are overlaid and depicted as the black population (Q8). CD56 gating was performed on single cells. (B) Compiled data on the percentage of gated subsets that are positive for CD16 (Q5 and Q6) and CD69 (Q6 and Q7). Patients (*n* = 9) are depicted as filled circles, and healthy controls (*n* = 5) are depicted as open circles. All data are presented as means ± standard deviations. *, *P* < 0.05, by Mann-Whitney *U* test, two-tailed.

IPA predicted robust cross talk between NK cells and moDCs in peripheral blood upon *ex vivo* ZIKV infection (Fig. 2 and 3). The IPA prediction that NK cells were activated in the peripheral blood of ZIKV-infected patients was, therefore, investigated by comprehensive immunophenotyping of blood samples taken from ZIKV-infected patients. These patients were recruited from the first endemic ZIKV outbreak in Singapore in 2016 (7, 14). Blood aliquots were obtained from ZIKV-infected patients (*n* = 9) during the acute disease phase (between 1 and 7 days post-illness onset [PIO]) and were subjected to a whole-blood staining protocol that targeted CD56^+^ cells, predominantly NK cells (15) (Fig. 4A). Blood from healthy donors (*n* = 5) was collected and processed in parallel as a control group. Gated cells were further grouped with the C-type lectin receptor CD94, giving three CD56^+^ populations: CD56^bright^ CD94^hi^, CD56^dim^ CD94^hi^, and CD56^dim^ CD94^lo^ (16). The activation status of these populations was then assessed based on the percentage of each subset expressing CD16 and CD69 (Fig. 4B). A higher level of CD16 was observed across all CD56^+^ subsets in ZIKV-infected patients than in the healthy controls. A higher percentage of the subsets also expressed CD69, a known cellular activation marker (17).

### CD14^+^ monocytes prime NK cell activity during ZIKV infection.

Given that peripheral NK cells were activated in ZIKV-infected patients and monocytes are precursors of MDMs, the functional relationship between monocytes and NK cells was assessed. CD14^+^ monocytes were depleted from human primary PBMCs, with an average efficiency of >95% (Fig. S2A). Lipopolysaccharide (LPS; 10 ng/ml) was used as a positive control to simulate priming of CD56^+^ CD94^+^ Lineage^−^ NK cells (Fig. S2B) by monocytes (18). A significant reduction in the activity of NK cells was observed when CD14-depleted PBMCs were stimulated with LPS compared to LPS stimulation of PBMCs containing CD14^+^ monocytes (Fig. S3A). This effect was evidenced by the reduced levels of the surface markers CD69, CD107a, and intracellular gamma interferon (IFN-γ) in depleted cells, verifying that this approach was an efficient strategy for investigating priming of NK cells by CD14^+^ monocytes.

10.1128/mSphereDirect.00120-18.2 FIG S2 Gating strategy of CD56^+^ CD94^+^ NK cells. (A) Fresh human PBMCs were subjected to CD14^+^ monocyte depletion. Flow cytometric plots from one representative donor are shown. A depletion efficiency of >95% was typically obtained. Monocytes are defined as lineage^+^ cells, with CD14, CD3, CD19, and CD20 included as lineage markers. (B) Live singlets were first gated from the stained PBMCs. CD45^+^ CD56^+^ cells were identified, and NK cells were subsequently gated out with CD94 and lineage markers—CD14, CD3, CD19, and CD20. NK cells are defined as CD56^+^ CD94^+^ Lineage^−^. Flow cytometric plots from one representative donor are shown. Download FIG S2, PDF file, 0.5 MB. Copyright © 2018 Lum et al. 2018 Lum et al. This content is distributed under the terms of the Creative Commons Attribution 4.0 International license.

10.1128/mSphereDirect.00120-18.3 FIG S3 Expression of surface markers by activated NK cells. LPS (10 ng/ml)-stimulated conditions. (A) Compiled percentages of CD69-, CD107a-, and IFN-γ-positive CD56^+^ CD94^+^ Lineage^−^ NK cells after LPS (10 ng/ml) stimulation. Expression levels are normalized to the respective mock sample (dotted line). Data shown were derived from seven healthy donors. (B) Total PBMCs and CD14-depleted PBMCs (2 × 10^6^ cells per infection) were infected with ZIKV at an MOI of 10 and harvested at 36 hpi. (B) Compiled percentages of NKG2D and NKG2A-positive CD94^+^ CD56^+^ Lineage^−^ NK cells normalized to the respective mock sample. (C) Comparison of the percentage of CD69-positive CD94^+^ CD56^+^ Lineage^−^ NK cells between mock-infected and ZIKV-infected full PBMCs and CD14-depleted PBMCs. Data shown were derived from seven donors. Data shown are presented as paired data. (D) The expression levels of CD69, CD107a, and IFN-γ on CD56^+^ CD94^+^ Lineage^−^ NK cells at 72 hpi. Expression levels are normalized to respective mock sample. Data were obtained from two donors. Lineage markers CD3, CD19, CD20, and CD14 have been included to rule out the presence of non-NK cells. All data are presented as means ± standard deviations. *, *P* < 0.05, by Mann-Whitney *U* test, two-tailed. Download FIG S3, PDF file, 0.2 MB. Copyright © 2018 Lum et al. 2018 Lum et al. This content is distributed under the terms of the Creative Commons Attribution 4.0 International license.

**FIG 5:**
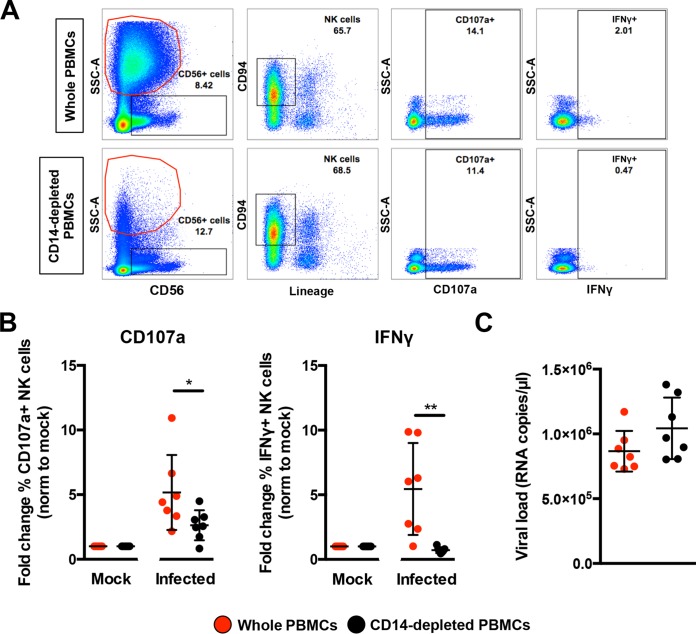
Role of monocytes in NK cell activity. Full PBMCs and CD14-depleted PBMCs (2 × 10^6^ cells per infection) were infected with Zika virus (ZIKV) at an MOI of 10 and harvested at 36 hpi. (A) Gating strategy of CD94^+^ CD56^+^ Lineage^−^ NK cells and their expression of CD69, CD107a, and IFN-γ. Plots from one representative donor are shown. The red circle indicates the presence or absence of CD14^+^ monocytes. (B) Compiled percentages of CD107a- and IFN-γ-positive NK cells (depicted in panel A) as normalized to the respective mock sample. (C) Viral load in the infected cells. Data shown were derived from seven donors. Lineage markers CD3, CD19, CD20, and CD14 have been included to rule out the presence of non-NK cells. All data are presented as means ± standard deviations. *, *P* < 0.05; **, *P* < 0.01, by Mann-Whitney *U* test, two-tailed. Viral load data were not statistically significant between the two conditions by Mann-Whitney *U* test, two-tailed.

PBMCs were then isolated from seven healthy donors and subjected to CD14 depletion before being either infected with ZIKV or stimulated with LPS in parallel to serve as a control to determine activation of NK cells. ZIKV infection in nondepleted PBMCs resulted in high levels of CD107a and IFN-γ (Fig. 5A) in CD56^+^ CD94^+^ Lineage^−^ NK cells at 36 hpi—an optimal time point to detect NK cell priming (19). The opposite effect, however, was observed in ZIKV-infected PBMCs depleted of CD14^+^ monocytes, as the levels of both CD107a and IFN-γ were significantly reduced (Fig. 5B). Although monocyte depletion did not affect the expression of NK cell activation receptor NKG2A or NKG2D, a general reduction in NKG2D-expressing NK cells was observed during ZIKV infection (Fig. S3B). Surprisingly, the activation marker CD69 was not increased upon ZIKV infection in this study (Fig. S3C). ZIKV viral loads were comparable between the two conditions (Fig. 5C). Interestingly, levels of CD107a and IFN-γ remained high at 72 hpi in nondepleted infected PBMCs compared to depleted infected PBMCs (Fig. S3D).

**FIG 6:**
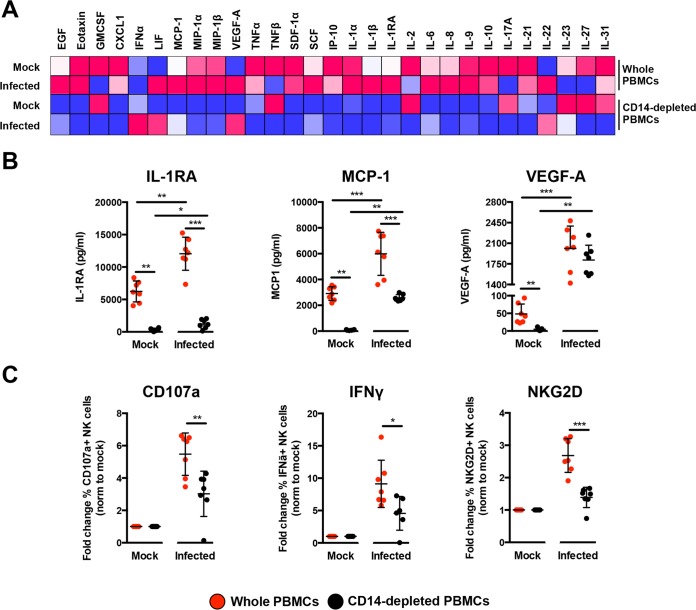
Immune profiling of ZIKV-infected PBMCs. (A) Immune mediators in the culture supernatant of ZIKV-infected PBMCs and CD14-depleted PBMCs were quantified with a 45-plex microbead assay. Concentrations were scaled between 0 and 1. (B) Bar charts of three cytokines, levels of which were significantly affected by both the depletion of CD14^+^ monocytes and ZIKV infection. (C) Stimulatory capacity of the culture supernatants was further evaluated with freshly isolated PBMCs. Culture supernatant was added in a ratio of 1:10, and cells were harvested at 36 h poststimulation. Compiled percentages of CD107a-, IFN-γ-, and NKG2D-positive CD94^+^ CD56^+^ NK cells are shown as normalized to the respective mock sample. Data displayed were derived from seven donors. Lineage markers CD3, CD19, CD20, and CD14 have been included to rule out the presence of non-NK cells. All data are presented as means ± standard deviations. *, *P* < 0.05; **, *P* < 0.01; ***, *P* < 0.001, by Mann-Whitney *U* test, two-tailed.

To delve further into the mechanism, the profile of secreted immune mediators from ZIKV-infected PBMCs was quantified using a 45-plex microbead-based immunoassay (20). Levels of 11 mediators were significantly affected by the depletion of CD14^+^ monocytes (Fig. 6A and S4A), while 8 mediators were affected upon ZIKV infection (Fig. S4B). Interestingly, depletion of CD14^+^ monocytes and ZIKV infection did not affect the levels of epidermal growth factor (EGF), interleukin 9 (IL-9), IL-17A, macrophage inflammatory protein 1α (MIP-1α), and MIP-1β (Fig. S4C). The effect of CD14^+^ monocyte depletion was observed in the levels of stem cell factor (SCF) and tumor necrosis factor alpha (TNF-α) only after ZIKV infection (Fig. S4D). Importantly, levels of monocyte chemoattractant protein 1 (MCP-1), IL-1RA, and vascular endothelial growth factor A (VEGF-A) were affected by both CD14^+^ monocyte depletion and ZIKV infection (Fig. 6B). To further investigate the capacity of the cytokine milieu to prime NK cells, freshly isolated human primary PBMCs were then treated with the same culture supernatants from ZIKV-infected PBMCs and CD14^+^ monocyte-depleted PBMCs. Stimulation with culture supernatant from ZIKV-infected nondepleted PBMCs led to a significant upregulation in expression of CD107a, IFN-γ, and NKG2D in the CD94^+^ CD56^+^ NK cells (Fig. 6C), confirming the importance of monocytes in NK cell priming during ZIKV infection. To rule out priming of NK cells by viruses present in the culture supernatant, a UV treatment procedure was performed to inactivate the virus prior to the stimulation assay. Expectedly, while UV inactivation successfully inactivated ZIKV (Fig. S5A), it also affected the quality of the cytokines and led to reduced priming of NK cells (Fig. S5B).

10.1128/mSphereDirect.00120-18.4 FIG S4 Quantification of immune mediators. Immune mediators in the culture supernatant of ZIKV-infected PBMCs and CD14-depleted PBMCs were quantified using a 45-plex microbead assay. Quantified immune mediators are grouped into four groups based on their profile: mediators affected by depletion of CD14^+^ monocytes (A), mediators affected by ZIKV infection (B), mediators not affected by both CD14^+^ monocyte depletion and ZIKV infection (C), and mediators affected by depletion of CD14^+^ monocytes only after ZIKV infection (D). Data displayed were derived from seven donors. All data are presented as means ± standard deviations. *, *P* < 0.05; **, *P* < 0.01; ***, *P* < 0.001, by Mann-Whitney *U* test, two-tailed. Download FIG S4, PDF file, 0.5 MB. Copyright © 2018 Lum et al. 2018 Lum et al. This content is distributed under the terms of the Creative Commons Attribution 4.0 International license.

10.1128/mSphereDirect.00120-18.5 FIG S5 UV inactivation of ZIKV and proteins. (A) Wild-type (WT) ZIKV was subjected to two different doses (1,000 or 100 mJ/cm^2^) of UV treatment across different durations. UV-treated ZIKV was subsequently used to infect HEK293T cells, and the amount of viral RNA load was determined at 48 hpi. Levels of viral RNA load are expressed as fold increase relative to the level of viral RNA load detected at 0 hpi with the WT ZIKV. Heat-inactivated (HI) ZIKV was included in parallel as a negative control. (B) Culture supernatants were UV treated (100 mJ/cm^2^ for 10 min), and their stimulatory capacity was further evaluated with freshly isolated PBMCs in a ratio of 1:10 (with fresh complete medium). Cells were harvested at 36 h poststimulation and evaluated for the percentage of CD107a-; IFN-γ-; and NKG2D-, NKG2A-, and CD69-positive CD94^+^ CD56^+^ NK cells. Data shown (*n* = 2) normalized to the respective mock sample. Lineage markers CD3, CD19, CD20, and CD14 have been included to rule out the presence of non-NK cells. All data are presented as means ± standard deviations. Download FIG S5, PDF file, 0.1 MB. Copyright © 2018 Lum et al. 2018 Lum et al. This content is distributed under the terms of the Creative Commons Attribution 4.0 International license.

## DISCUSSION

Myeloid cells are targets of active ZIKV infection (6–9, 21–23) and can elicit immune responses with detrimental outcomes (6, 8). Both monocytes and macrophages exhibit extensive heterogeneity (24, 25). While it is difficult to obtain tissue-resident macrophages for experimental purposes, human blood is a readily accessible, valuable source of these cells. Transcriptomic profiling of *ex vivo* human blood monocytes and MDMs has revealed marked differences between these cell types (26, 27). In this study, human primary monocytes were naturally differentiated into MDMs without any bias for an M1 or M2 macrophage phenotype (28). Given that these cells are targets of ZIKV infection (8), investigations into their cellular immune responses during infection will open avenues to exploit their function for therapeutic benefits.

The level of ZIKV infection (as assessed by the amount of ZIKV antigen and genome copy number) was higher in MDMs than monocytes, which corroborates previous observations (8). Transcriptomic differences between monocytes and MDMs (26, 27) would be a plausible explanation for the differential susceptibility of these cells to ZIKV infection. It is also noteworthy that higher ZIKV infection levels were found in purified primary cell populations than in PBMCs, perhaps due to the presence of other immune subsets in PBMCs that may dampen the overall infection level. ZIKV RNA was detected at the two time points, 24 and 72 hpi, and the virus was present as quasispecies postinfection in human primary myeloid cells. The virus consensus sequence and minor variant mapping revealed an overrepresentation of transition mutations at highly variable nucleotide positions in the sequence reads. The proportion of these minor variants indicated a shift toward becoming major variants. A recent study that sequenced ZIKV genomes isolated from infected patients provided important information pertaining to ZIKV transmission (29). These data highlighted the degree of divergence in sequenced genomes and placed further emphasis on understanding virus evolution and transmission effectiveness (30). As not all recovered ZIKV RNA samples contained the same mutations, it will be interesting to determine how different host immune responses can lead to ZIKV quasispecies that acquire different combinations of mutations.

ZIKV infection led to the differential abundance of host transcripts mapping to numerous cellular genes in MDMs but not in monocytes, likely due to higher levels of infection observed in MDMs. Furthermore, it has been reported that different donors could account for significant differences in cellular responses (31, 32). However, this differential effect does not necessarily signify that ZIKV-infected monocytes do not elicit any cellular response to infection but rather that the differences were not measurable by RNA-seq at the read depths used in this analysis. In fact, transcript abundances of numerous genes were different between the mock- and ZIKV-infected monocytes; however, the statistical threshold of an FDR of <0.05 was not reached and thus these findings were excluded from further analyses. Using IPA data mining, these differentially expressed genes were involved in 133 and 63 canonical cellular pathways (27 of them being shared) in MDMs at 24 and 72 hpi, respectively. The reduced number of cellular pathways identified in ZIKV-infected MDMs at the later 72-hpi time point suggests that certain cellular functions may be shut down postinfection (33). This effect could signify that (i) the host cells conserve energy to focus only on essential pathways for survival and/or (ii) the host cells have succumbed to ZIKV infection, which leads to transcriptional shutdown in host cells.

Unsurprisingly, the IFN response was the most highly expressed signaling pathway of these common pathways at both time points because of the virus trigger (34). This observation was further complemented by the presence of few other IFN-related pathways. Observations were found for the next two most expressed pathways—pathogenesis of multiple sclerosis and cross talk between NK cells and moDCs—both of which involve NK cells. Although ZIKV infection has not been previously associated with multiple sclerosis due to the relatively new disease spectrum, other viral infections such as those with Epstein-Barr virus (35) and measles virus (36) have been linked.

CXCL9, CXCL10, CXCL11, and CCL5 (identified as the top genes in the pathway) are chemokines known to stimulate NK cell activation (37, 38). The increased transcript abundance of these immune mediators, coupled with others such as IL-15, is a strong indication that ZIKV-infected macrophages are primed to “communicate” with NK cells. Other recent studies have also provided evidence of cross talk between macrophages and NK cells (18). The increased abundance of TNFSF10 and Fas transcripts in ZIKV-infected MDMs could indicate priming of NK cell-mediated apoptosis (39). Interestingly, levels of typical NK cell-activating cytokines, such as IL-12 (40, 41) and IL-18 (42, 43), were not differentially expressed in this study. However, mRNA levels of IL-23 and IL-27, two cytokines belonging to the family of IL-12 (44) with roles in NK cell activation (45, 46), were increased.

Immunophenotyping of whole-blood samples from ZIKV-infected patients revealed the presence of CD69^+^ CD56^+^ immune cells (predominantly the CD56^+^ NK cells) (15), suggesting the possible priming of NK cells in ZIKV infection. Similar observations were also reported in dengue virus (DENV) patients (47), as well as in volunteers vaccinated with the yellow fever virus (YFV) vaccine (48). The involvement of NK cells was thus explored *ex vivo* in human primary PBMCs. Interestingly, *ex vivo* culture alone led to an increase in the basal expression level of CD69 in CD56^+^ CD94^+^ NK cells, as previously reported (49). Furthermore, ZIKV infection resulted in reduced levels of CD69, which is a phenomenon also reported for the flavivirus tick-borne encephalitis virus infection in healthy donor NK cells (50). Moreover, NK cells behave differently *ex vivo* and *in vivo* (51), which may explain the different levels of CD69 detected in patients and in *ex vivo* ZIKV-infected NK cells. It was also reported in CD69-deficient mice that the activity of NK cells remains functional (52). High levels of key NK activation markers, including the degranulation marker CD107a and intracellular cytokine IFN-γ, indicate the higher activation status of NK cells. The activity of NK cells was directly dependent on the presence of CD14^+^ monocytes. ZIKV infection of PBMCs depleted of CD14^+^ monocytes significantly downregulated the expression of the various NK cell markers, demonstrating the functional role of monocytes as one of the key players for NK cell stimulation. The data presented in this study are further supported by a recent publication in which ZIKV patients had high levels of IL-18, TNF-α, and IFN-γ (20)—immune mediators associated with NK cell function. The use of SJL mice, which lack NK cells (53), as a model of ZIKV infection also suggested a protective role for these immune cells given that these animals succumbed to cortical malformations (54). Likewise, the NK cell-mediated immune response was significantly increased in healthy volunteers receiving a YFV vaccination (55). Thus, the role of NK cells during ZIKV infection should be explored.

Interestingly, multiplex quantification of secreted immune mediators from *ex vivo* ZIKV-infected PBMCs provided an alternate perspective. IL-18 and IFN-γ, two NK cell-related cytokines, were below the detection limit. However, freshly isolated PBMCs stimulated with culture supernatants from ZIKV-infected PBMCs resulted in increased priming of NK cells, clearly indicating that the concoction of immune mediators is capable of driving NK cell activation.

Nonetheless, the, depletion of CD14^+^ monocytes would abrogate this activation as observed by the low levels of MCP-1, IL-1RA, VEGF-A, eotaxin, growth-related oncogene alpha (GROα), IFN-α, stromal cell-derived factor 1α (SDF-1α), IFN-γ-induced protein 10 (IP-10), IL-6, IL-1α, IL-1β, IL-8, IL-21, and IL-10. The reduced levels of MCP-1 could also have a detrimental effect on NK cell recruitment and priming (37, 56), although MCP-1 and VEGF-A have been reported to drive the production of each other (57–59). The high levels of secreted IL-1RA from ZIKV-infected PBMCs could also have participated in the increased priming of NK cells, as IL-1RA is known to potentiate the effect of IL-2 stimulation of NK cells (60). Thus, the loss of detectable IL-2 after ZIKV infection in CD14-depleted PBMCs would further reduce NK cell priming. The presence of other immune mediators, such as IL-6, IL-8, IL-10, IP-10, SDF-1α, GROα, IL-1α, and IL-1β, in ZIKV-infected nondepleted PBMCs would further provide an inflammatory condition for cellular activation. While the levels of these immune mediators have been reported to be high in ZIKV patients (20), IL-10 and IP-10 have been demonstrated to contribute to cytolysis and activation of NK cells (37, 61). Levels of leukemia inhibitory factor (LIF) (62), IL-22 (63), and IL-31 (64) were high upon ZIKV infection, indicating their roles in regulating T cells during ZIKV infection (65). T cells could regulate NK cell activity (66), and monocytes could indirectly mediate NK cell functions through the T lymphocytes. Though it was not exhibited in this work, monocytes are known to physically “communicate” with T cells, regulating their activities in the process (67). This is thought to occur via the interaction between CD80/86 and CD28 expressed on the infected monocytes and T cells, respectively (68). Likewise, monocytes could also activate NK cells via physical interactions (69). Interestingly, it was recently reported that NK cells were activated by monocyte-derived dendritic cells in a contact-dependent manner during DENV infection (70). This further highlights the complexity of immune cell interactions in different contexts.

To conclude, through a systematic investigative workflow combining approaches exploring host cell transcriptomes and immunoproteomes, it was demonstrated that monocytes and macrophages do not act alone but in conjunction with other immune cells to orchestrate a series of host immune responses and drive disease progression. As such, a comprehensive understanding of immune cell interaction will have important clinical implications for the design of novel therapeutics that can either dampen down or enhance a response as appropriate.

## MATERIALS AND METHODS

### Ethics approval and consent to participate.

Whole-blood samples were collected from ZIKV-infected patients who were referred to the Communicable Disease Centre, Tan Tock Seng Hospital, Singapore. Blood was obtained from patients who provided written informed consent. The study protocol was approved by the SingHealth Centralized Institutional Review Board (CIRB; reference no. 2016/2219). Blood samples were collected from healthy donors with written consent in accordance with guidelines from the Health Sciences Authority of Singapore (study approval number NUS IRB 10-250).

### Patient whole-blood samples.

This study utilized whole-blood samples obtained from patients (*n* = 9) admitted to the Communicable Disease Centre at Tan Tock Seng Hospital, Singapore, from 27 August to 18 October 2016. Samples included in this study were collected during the acute phase (1 to 7 days post-illness onset [PIO]) of ZIKV infection. These patients were confirmed to be infected with ZIKV by reverse transcription-PCR (RT-PCR) performed on serum and urine samples obtained during their first visit to the clinic. Patients were screened negative for DENV exposure by RT-PCR and serology. Whole-blood samples were collected in EDTA Vacutainer tubes (Becton, Dickinson). Whole-blood samples were also obtained from healthy volunteers (*n* = 5) as controls, which were confirmed to be negative for ZIKV RNA by RT-PCR.

### Virus preparation.

The ZIKV strain (GenBank accession number KJ776791.2) used in this study was originally isolated from the French Polynesia outbreak in 2013 (71). The virus was propagated as previously described (8). Briefly, the virus was propagated by multiple passages in Vero-E6 cells (ATCC; CRL-1587) and precleared by centrifugation before storage at −80°C. The ZIKV stock contained approximately 1.2 × 10^7^ PFU/ml of infectious virus when titrated on Vero-E6 cells and approximately 3.5 × 10^9^ ZIKV viral RNA copies/ml by the quantitative RT-PCR method described below. The virus titer was determined using standard plaque assays with Vero-E6 cells. Vero-E6 cells were regularly tested for mycoplasma contamination and were grown and passaged in Dulbecco’s modified Eagle’s medium (DMEM; HyClone) supplemented with 10% (vol/vol) fetal bovine serum (FBS). UV inactivation of ZIKV was performed with the CL-1000 UV cross-linker (UVP) at an intensity of 100 mJ/cm^2^ for 10 min.

### Isolation and depletion of monocytes from human PBMCs.

Monocytes were prepared from fresh human PBMCs as previously described (8) and by gradient centrifugation using Ficoll-Paque density gradient medium (GE Healthcare). Subsequently, monocytes were isolated using an indirect magnetic labeling system (monocyte isolation kit II; Miltenyi Biotec). A direct magnetic labeling system (human CD14^+^ monocyte isolation kit 2; StemCell) was used for depletion of monocytes from PBMCs. The manufacturers’ protocols were strictly adhered to for these procedures.

### Differentiation of monocytes into MDMs.

Isolated monocytes were differentiated into MDMs by plating in complete Iscove’s modified Dulbecco’s medium (IMDM) (HyClone) supplemented with 10% (vol/vol) heat-inactivated human serum (HS) (Sigma-Aldrich), which was replaced every 2 days. ZIKV infections were performed on monocytes and MDMs 5 days later, as described below.

### Virus infection.

ZIKV infections in monocytes and MDMs (*n* = 5 each) were performed at a multiplicity of infection (MOI) of 10. Each infection mixture consisted of a virus suspension prepared in serum-free IMDM (HyClone). The cells were incubated with the infection mixture at 37°C and allowed to adsorb for 2 h with intermittent shaking before the virus inoculum was removed and replaced with complete IMDM supplemented with 10% (vol/vol) HS (Sigma-Aldrich). Cells were incubated at 37°C until harvesting at 24 and 72 hpi. The harvested cells for downstream total RNA isolation were stored at −80°C. A total of 140 µl of the infected-cell suspension was used to quantify the viral load. For assessment of monocyte function in NK cell activation during ZIKV infection, total human PBMCs (*n* = 7) and donor-corresponding CD14-depleted PBMCs (*n* = 7) were infected with ZIKV at an MOI of 10. In parallel, both PBMC fractions were stimulated with 10 ng/ml lipopolysaccharide (LPS; Sigma) as a positive control to measure NK cell activation. Cells were subsequently treated with 1× brefeldin (eBioscience) and stained with CD107a (BD Pharmingen) 6 h before harvesting at 36 hpi. The viral load was quantified from 140 µl of the infected-cell suspension. Negative controls (*n* = 7) were cells undergoing the same infection conditions in the absence of infectious ZIKV particles. These controls are referred to as mock-infected samples.

### PBMC stimulation assay.

Fresh PBMCs were isolated as described above and subjected to stimulation with ZIKV-infected culture supernatants (*n* = 7) in a final ration of 1:10 in fresh IMDM (HyClone) supplemented with 10% (vol/vol) HS (Sigma-Aldrich). Cells were subsequently treated with 1× brefeldin (eBioscience) and stained with CD107a (BD Pharmingen) 6 h before harvesting at 36 h for downstream antibody staining.

### Viral RNA extraction and viral load analysis.

Viral RNA was extracted using a QIAamp viral RNA minikit (Qiagen), according to the manufacturer’s instructions. Quantification of ZIKV NS5 RNA was determined by quantitative real-time PCR (qRT-PCR) TaqMan assay (72) using a QuantiTect Probe RT-PCR kit (Qiagen) in a 12.5-µl reaction volume. All reactions were performed on a 7900HT Fast real-time PCR system machine (Applied Biosciences).

### Total RNA extraction.

Total RNA was extracted using an RNeasy minikit (Qiagen) according to the manufacturer’s instructions. The extracted total RNA was quantified on a NanoDrop 1000 spectrophotometer (Thermo Fisher Scientific).

### Flow cytometry and antibodies.

Detection of ZIKV antigen was carried out in a two-step indirect intracellular labeling process. Briefly, harvested cells were first fixed and permeabilized with fluorescence-activated cell sorting (FACS) lysing solution (BD Biosciences) and FACS permeabilization solution 2 (BD Biosciences), respectively. Antigen staining was then performed with a flavivirus-specific mouse monoclonal antibody (clone 4G2) (Millipore) followed by secondary staining with a goat anti-mouse IgG F(ab′)_2_ antibody (Invitrogen). Cells were then specifically stained for the surface markers CD45 and CD14 (for ZIKV-infected monocytes and MDMs). Dead cells were excluded by staining with the LIVE/DEAD Fixable Aqua dead cell stain kit (Life Technologies). For PBMCs, surface markers CD45, CD14, CD3, CD19, and CD56 were stained prior to intracellular staining (for ZIKV-infected PBMCs). For patient samples, 100 μl of whole blood was stained for the surface markers CD56, CD94, CD16, CD69, CD107a, NKG2D, and NKG2A. The stained cells were subsequently incubated with FACS lysing solution (BD Biosciences) to lyse the red blood cells. Lymphocytes were first gated to exclude the neutrophils. Subsequently, CD56^+^ cells were first identified and further defined with the CD94 surface marker to give three other subsets—CD56^bright^ CD94^hi^, CD56^dim^ CD94^hi^, and CD56^dim^ CD94^lo^ (16). To specifically assess NK cell activity *ex vivo*, PBMC fractions were stained for CD107a and various lineage markers (CD3, CD19, CD20, and CD14) (15) in addition to the panel of antibodies used for patient whole-blood staining. The usage of lineage markers excludes the presence of non-NK cells in the ensuing analysis. Stained PBMCs were fixed and permeabilized as described above before intracellular staining of ZIKV antigen and IFN-γ. All gatings were performed on single cells.

All antibodies used were mouse anti-human antibodies and were obtained from BD Pharmingen (CD3, CD19, CD20, CD14, CD69, CD56, CD94, NKG2D, CD107a, and IFN-γ), BioLegend (CD16 and CD45), and Miltenyi Biotec (NKG2A). Data were acquired on a Fortessa flow cytometer (BD Biosciences) with BD FACSDiva software. Data analysis was performed using FlowJo version 9.3.2 software (Tree Star, Inc.).

### Cytokine quantification using microbead-based immunoassay and data analyses.

Cytokine levels in supernatant obtained from mock- and ZIKV-infected PBMCs were measured simultaneously using the ProcartaPlex immunoassay (Thermo Fisher Scientific) detecting 45 secreted cytokines, chemokines, and growth factors, including brain-derived neurotropic factor (BDNF); eotaxin/CCL11; epidermal growth factor (EGF); fibroblast growth factor 2 (FGF-2); granulocyte-macrophage colony-stimulating factor (GM-CSF); growth-related oncogene alpha (GROα)/CXCL1; hepatocyte growth factor (HGF); nerve growth factor (NGF) beta (73); leukemia inhibitory factor (10); alpha interferon (IFN-α); IFN-γ; interleukin-1β (IL-1β); IL-1α; IL-1RA; IL-2; IL-4; IL-5; IL-6; IL-7; IL-8/CXCL8; IL-9; IL-10; IL-12p70; IL-13; IL-15; IL-17A; IL-18; IL-21; IL-22; IL-23; IL-27; IL-31; gamma interferon-induced protein 10 (IP-10)/CXCL10; monocyte chemoattractant protein (MCP-1/CCL2); macrophage inflammatory protein 1α (MIP-1α)/CCL3; MIP-1β/CCL4; regulated on activation, normal T cell expressed and secreted (RANTES)/CCL5; stromal cell-derived factor 1α (SDF-1α)/CXCL12; tumor necrosis factor alpha (TNF-α); TNF-β/lymphotoxin alpha (LTA); platelet-derived growth factor (PDGF)-BB; placental growth factor (PLGF); stem cell factor (SCF); vascular endothelial growth factor A (VEGF-A); and VEGF-D. Preparation of samples and reagents and immunoassay procedures were performed according to manufacturers’ instructions. Data were acquired using a Luminex FlexMap three-dimensional (3D) instrument (Millipore) and analyzed using Bio-Plex Manager 6.0 software (Bio-Rad) based on standard curves plotted through a five-parameter logistic curve setting. Levels of BDNF, FGF-2, HGF, nerve growth factor (NGF), IFN-γ, IL-4, IL-5, IL-7, IL-12p70, IL-13, IL-15, IL-18, RANTES, PDGF-BB, PLGF, and VEGF-D were below detection limit and excluded for further analysis. Hierarchical clustering was done using TM4-MeV (http://mev.tm4.org/).

### RNA-seq and differential gene expression analysis.

The general approach to RNA-seq and differential expression has been previously described (10, 74) and is detailed in brief below.

### RNA-seq.

RNA samples were treated with DNase using an Ambion Turbo DNA-free kit (Ambion) and then purified using AMPure XP beads (Agencourt). The DNase-treated RNA (2 µg) underwent Ribo-Zero treatment using an Epicentre Ribo-Zero Gold kit (human/rat/mouse) (Epicentre) and was repurified on AMPure XP beads. Successful RNA depletion was verified using a Qubit (Thermo Fisher Scientific) and an Agilent 2100 Bioanalyzer (Agilent), and all of the depleted RNA was used as input material for the ScriptSeq v2 RNA-seq library preparation protocol. RNA was amplified for 14 cycles, and the libraries were purified on AMPure XP beads. Each library was quantified using Qubit, and the size distribution was assessed using the AATI fragment analyzer (Advanced Analytical). These final libraries were pooled in equimolar amounts using the Qubit and fragment analyzer data. The quantity and quality of each pool were assessed by the fragment analyzer and subsequently by quantitative PCR (qPCR) using the Illumina library quantification kit (Kapa Biosystems) on a Light Cycler LC480II (Roche) according to the manufacturer’s instructions. The template DNA was denatured according to the protocol described in the Illumina cBot user guide and loaded at a 12 pM concentration. Sequencing was carried out on three lanes of an Illumina HiSeq 2500 with version 4 chemistry, generating 2- by 125-bp paired-end reads.

### Bioinformatics analysis.

Briefly, base calling and demultiplexing of indexed reads were performed using CASAVA version 1.8.2 (Illumina) to produce 30 samples from the five lanes of sequence data in fastq format. The raw fastq files were trimmed to remove the Illumina adapter sequences using Cutadapt version 1.2.1 (75). The option “-O 3” was set so that the 3′ end of any read that matched the adapter sequence by ≥3 bp was removed. The reads were further trimmed to remove low-quality bases using Sickle version 1.200 with a minimum window quality score of 20. After trimming, reads of <50 bp were removed. If both reads from a pair passed this filter, each read was included in the R1 (forward reads) or R2 (reverse reads) file. If only one read of a read pair passed this filter, it was included in the R0 (unpaired reads) file. The reference genome used for alignment was the human reference genome assembly GRCh38, downloadable from the Ensembl ftp site. The reference annotation was downloaded from the Ensembl ftp site (ftp://ftp.ensembl.org/pub/release-77/gtf/homo_sapiens/Homo_sapiens.GRCh38.77.gtf.gz). The annotated file contained 63,152 genes. R1/R2 read pairs were mapped to the reference sequence using TopHat2 version 2.1.0 (76), which employs the mapper Bowtie 2 version 2.0.10 (77).

### Differential gene expression and functional analysis.

Mapped reads were further analyzed using edgeR version 3.3 (78) to calculate normalized counts per million (CPM), identify genes differentially expressed between infected and mock-infected conditions, and compare infected conditions with each other. Correlation and principal-component analysis (PCA) plots were created in RStudio. Heat maps were generated using GENE-E (Broad Institute; https://software.broadinstitute.org/GENE-E/). IPA was used for gene ontology and pathway analysis. The *P* value associated with each identified canonical pathway was calculated by Fisher’s exact test (right-tailed). The presence of the 27 common canonical pathways was illustrated in a heat map generated by hierarchical clustering using TM4-MeV (79).

### Identification of ZIKV variants.

Bowtie 2 (77) was used to determine the mean sequence coverage. Here, 12 of the 41 samples (including the inoculum) had a mean coverage of >10 following alignment with the ZIKV reference genome (GenBank accession number KJ776791.2) used in this study. The frequencies of minor variants were calculated using QuasiRecomb (80). Sequences of individual viral proteins were compared to the Protein Data Bank using the online NCBI Protein BLAST server (https://blast.ncbi.nlm.nih.gov/Blast.cgi?PAGE=Proteins).
