# Supplementary figures and images for: Zika Virus Infection Preferentially Counterbalances Human Peripheral Monocyte and/or NK Cell Activity
Source: mSphere. 2018 Mar 28;3(2):e00120-18. doi: 10.1128/mSphereDirect.00120-18 (PMC5874443; doi:10.1128/mSphereDirect.00120-18)

**A**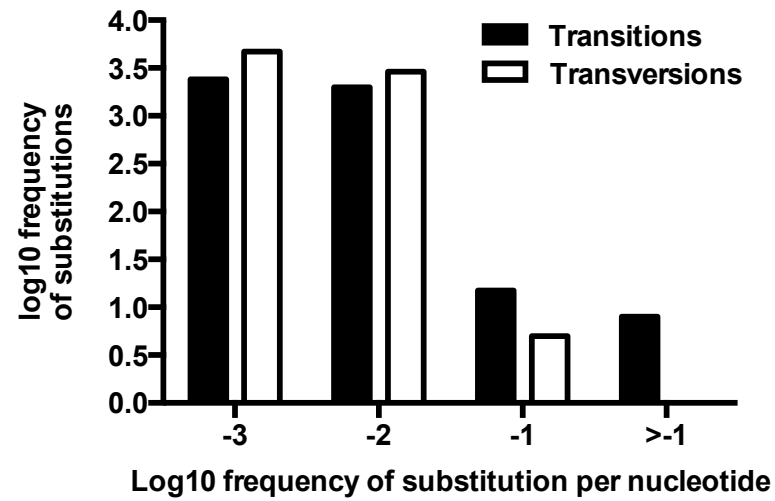**B**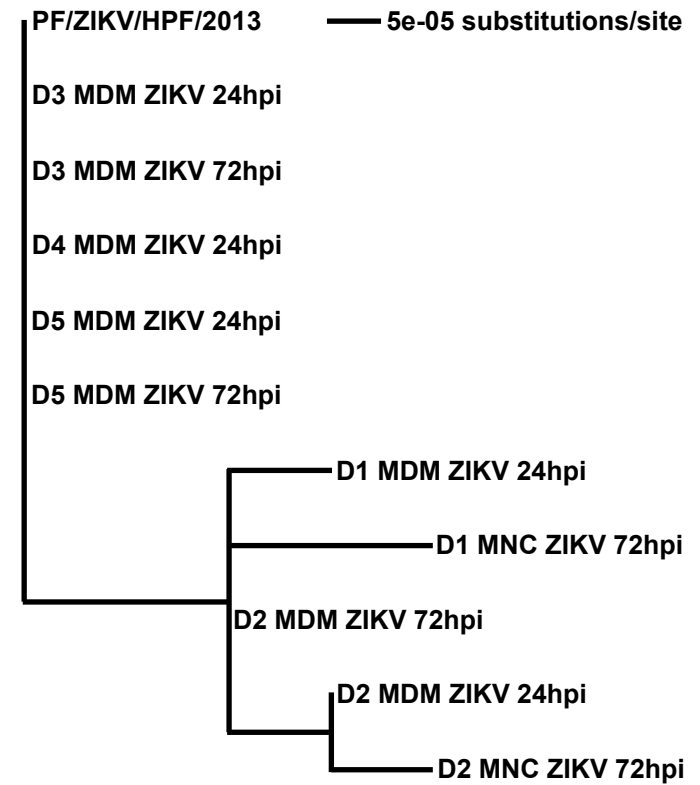

Supplemental Figure 1

Supplement: FIG S1 [file sph002182504sf1.pdf]

**A**

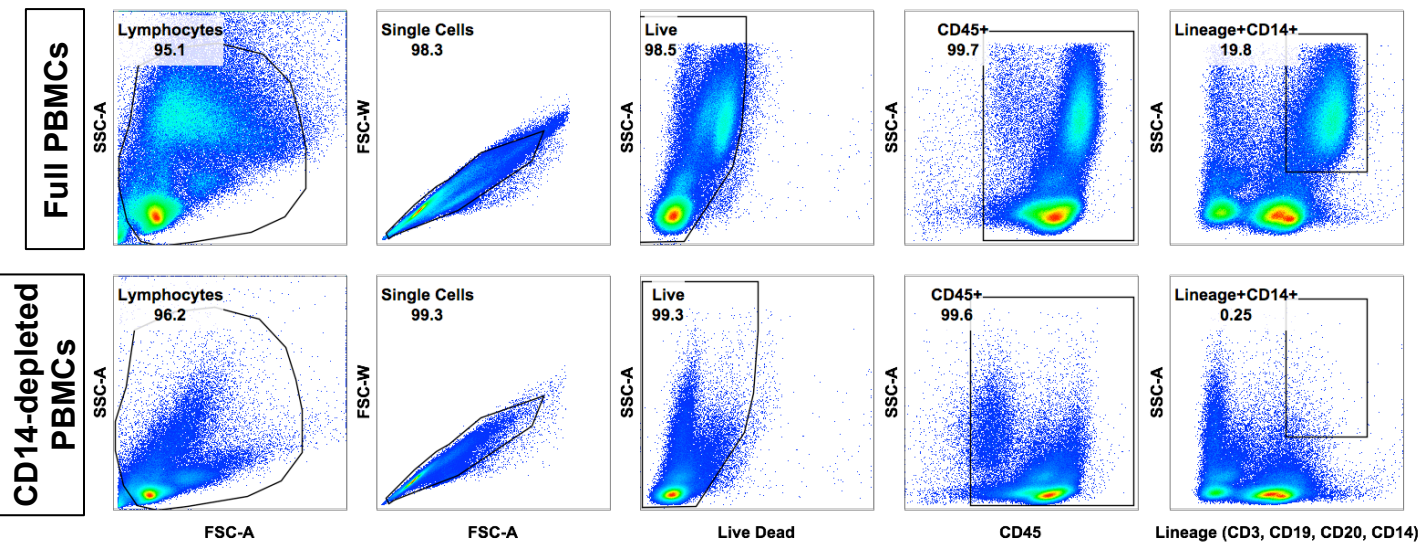

**B**

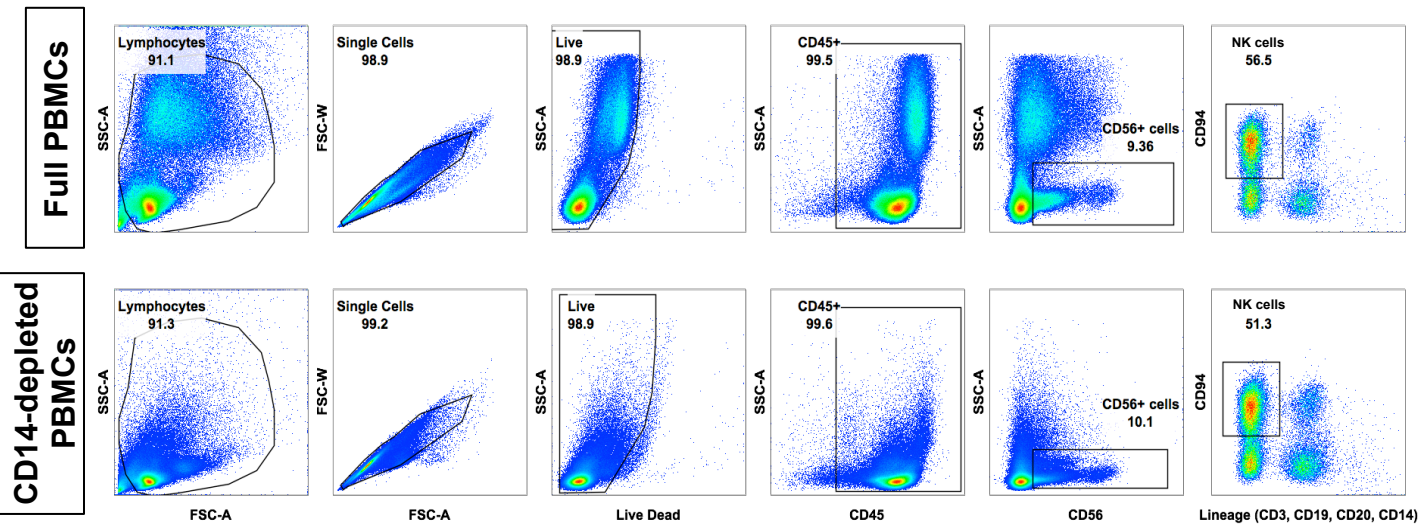

Supplemental Figure 2

Supplement: FIG S2 [file sph002182504sf2.pdf]

**A**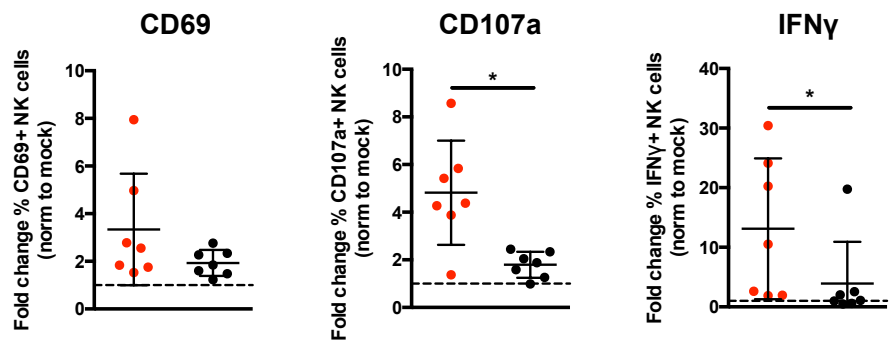**B**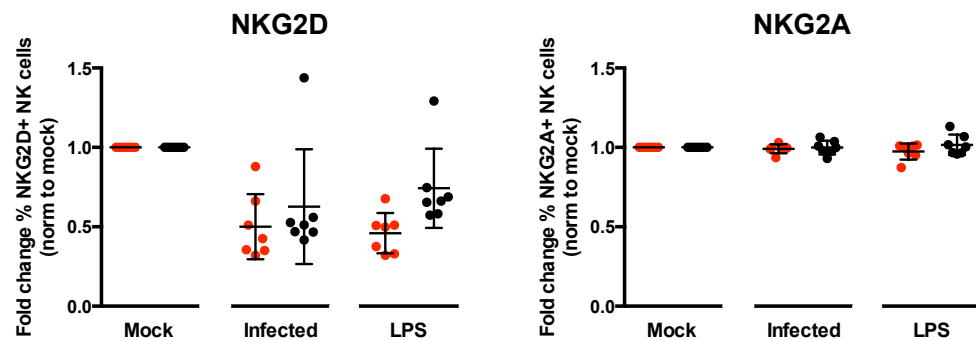**C**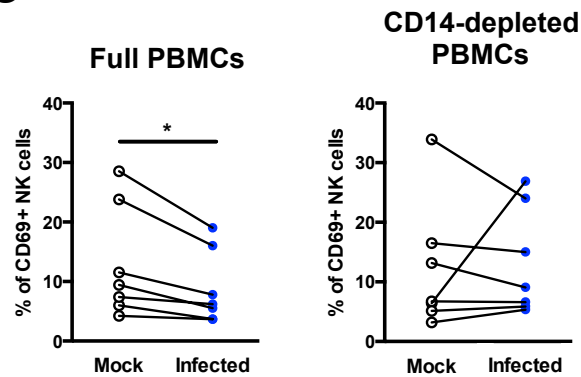**D**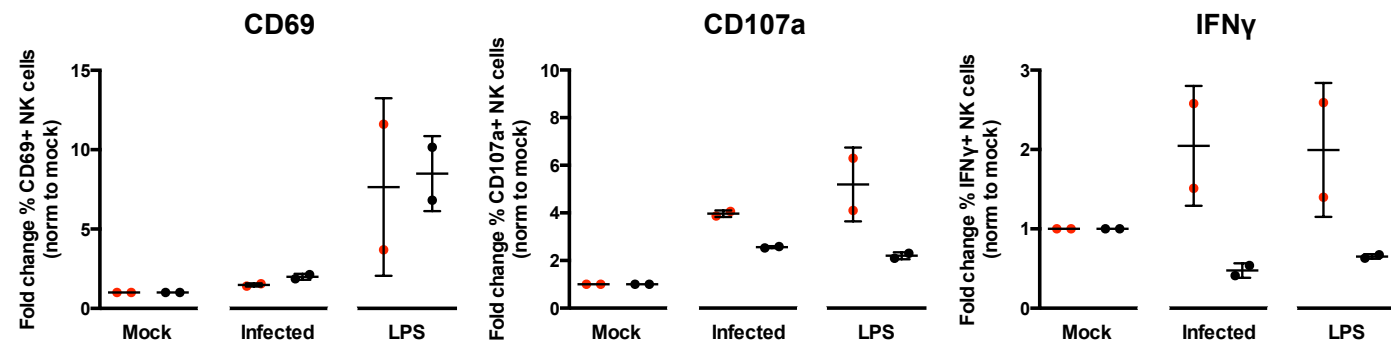

Supplemental Figure 3

Supplement: FIG S3 [file sph002182504sf3.pdf]

**A**

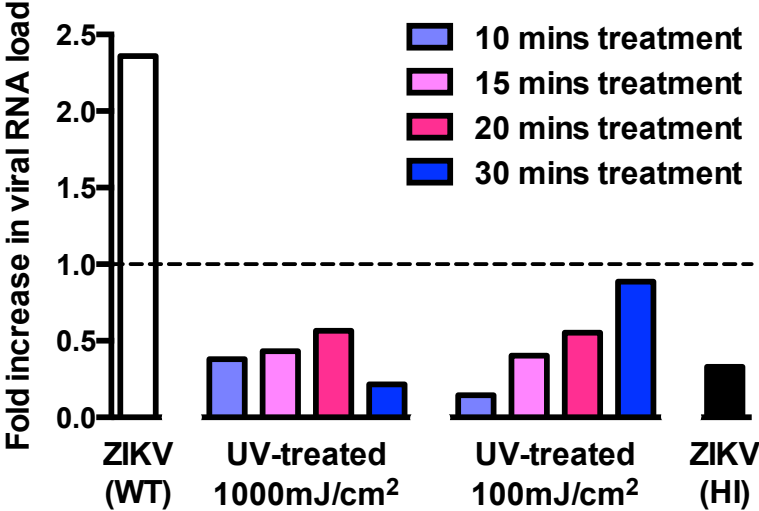

**B**

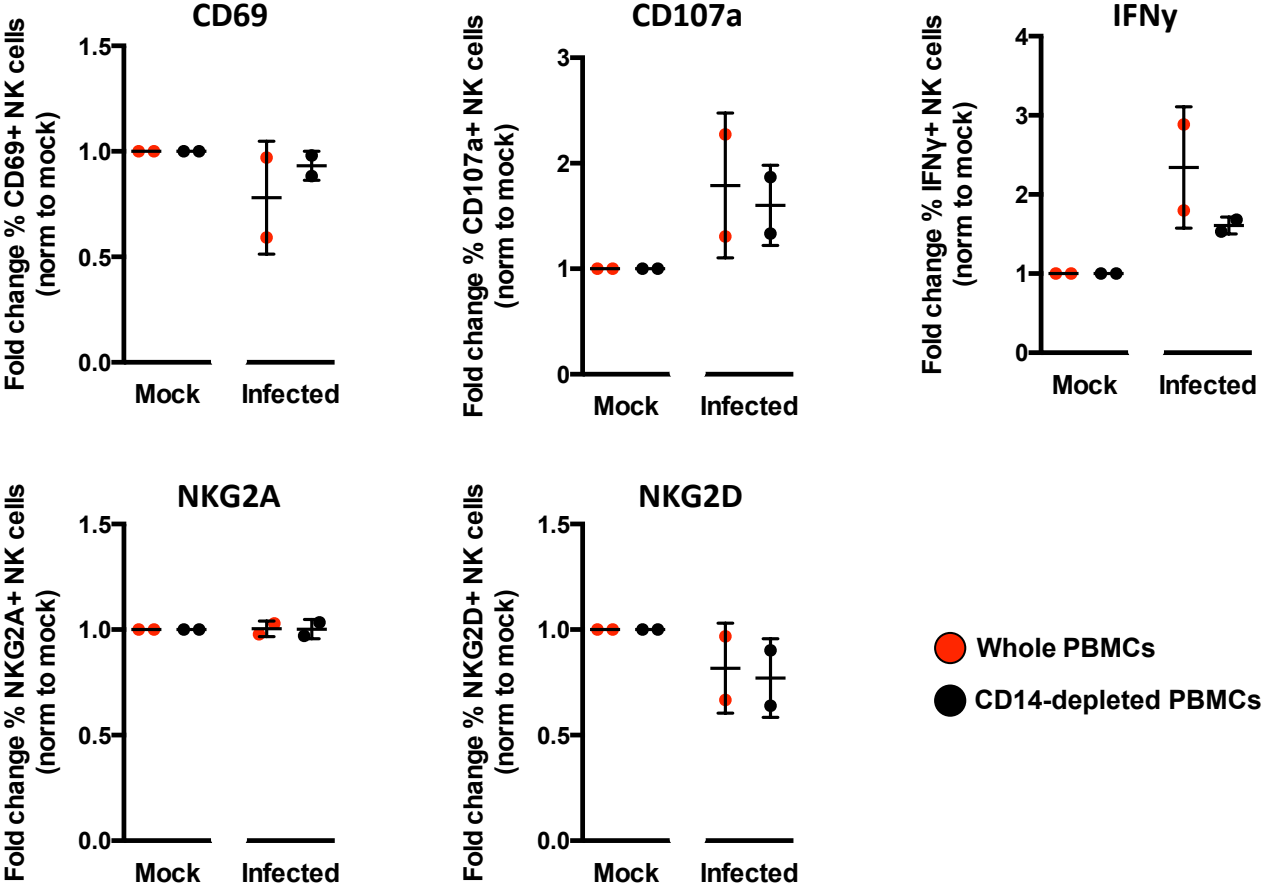

Supplemental Figure 5

Supplement: FIG S5 [file sph002182504sf5.pdf]
