# Supplementary material for: Zika Virus Infection Preferentially Counterbalances Human Peripheral Monocyte and/or NK Cell Activity
Source: mSphere. 2018 Mar 28;3(2):e00120-18. doi: 10.1128/mSphereDirect.00120-18 (PMC5874443; doi:10.1128/mSphereDirect.00120-18)
Supplement: TABLE S1 [file sph002182504st1.pdf]

Supplemental Table S1: Summary of nucleotide differences at specific genome positions[1]

Position in consensus sequence[2]	Nucleotide difference	Sample(s)
1904	g → a	D1 MNC ZIKV 72hpi
2673	t → c	D2 MDM ZIKV 24hpi<br>D2 MNC ZIKV 72hpi
2815	t	D1 MDM ZIKV 24hpi<br>D2 MDM ZIKV 24hpi<br>D2 MDM ZIKV 72hpi<br>D1 MNC ZIKV 72hpi<br>D2 MNC ZIKV 72hpi
	c	D3 MDM ZIKV 24hpi<br>D3 MDM ZIKV 72hpi<br>D4 MDM ZIKV 24hpi<br>D5 MDM ZIKV 24hpi<br>D5 MDM ZIKV 72hpi<br>PF/ZIKV/HPF/2013
4211	a	D3 MDM ZIKV 24hpi<br>D3 MDM ZIKV 72hpi<br>D4 MDM ZIKV 24hpi<br>D5 MDM ZIKV 24hpi<br>D5 MDM ZIKV 72hpi<br>PF/ZIKV/HPF/2013
	g	D1 MDM ZIKV 24hpi<br>D2 MDM ZIKV 24hpi<br>D2 MDM ZIKV 72hpi<br>D1 MNC ZIKV 72hpi<br>D2 MNC ZIKV 72hpi
10253	t → c	D1 MDM ZIKV 24hpi<br>D2 MNC ZIKV 72hpi
10472	t → c	D1 MNC ZIKV 72hpi

[1] The phylogenetic tree shown in Figure 3B revealed specific nucleotide differences at 6 different positions within the consensus sequence. All samples included had a mean coverage of greater than 10. PF/ZIKV/HPF/2013 represents the virus used for the infection and therefore, denoted as the reference sample in this analysis. Abbreviations: MNC, monocytes; MDM, monocyte-derived macrophage. [2]Note that minor variant file numbering of positions starts at 0 rather than 1.
