# Supplementary material for: Zika Virus Infection Preferentially Counterbalances Human Peripheral Monocyte and/or NK Cell Activity
Source: mSphere. 2018 Mar 28;3(2):e00120-18. doi: 10.1128/mSphereDirect.00120-18 (PMC5874443; doi:10.1128/mSphereDirect.00120-18)
Supplement: TABLE S2 [file sph002182504st2.pdf]

Supplemental Table S2: Selected nucleotide positions from the minor variants file of the inoculum[1]

Position in consensus sequence[2]	Frequency of minor nucleotide variants				
	A	C	G	T(U)	D (A/G/T)
1904	0.11324	0	0.88601	0.00074	0
2673	0.00681	0.11779	0.000619	0.87476	0
2815	0.00876	0.64463	0	0.34659	0
4211	0.65122	0	0.34815	0.000626	0
10253	0.000891	0.11229	0	0.8868	0
10472	0.00108	0.00543	0.00108	0.99239	0

[1]Table showing the frequency distribution of minor nucleotide variants at six positions in the consensus sequence. Major variant (i.e. the consensus nucleotide) at each position is indicated by the nucleotide with the highest frequency. [2]Note that minor variant file numbering of positions starts at 0 rather than 1.
