# Supplementary material for: Zika Virus Infection Preferentially Counterbalances Human Peripheral Monocyte and/or NK Cell Activity
Source: mSphere. 2018 Mar 28;3(2):e00120-18. doi: 10.1128/mSphereDirect.00120-18 (PMC5874443; doi:10.1128/mSphereDirect.00120-18)
Supplement: FIG S4 [file sph002182504sf4.pdf]

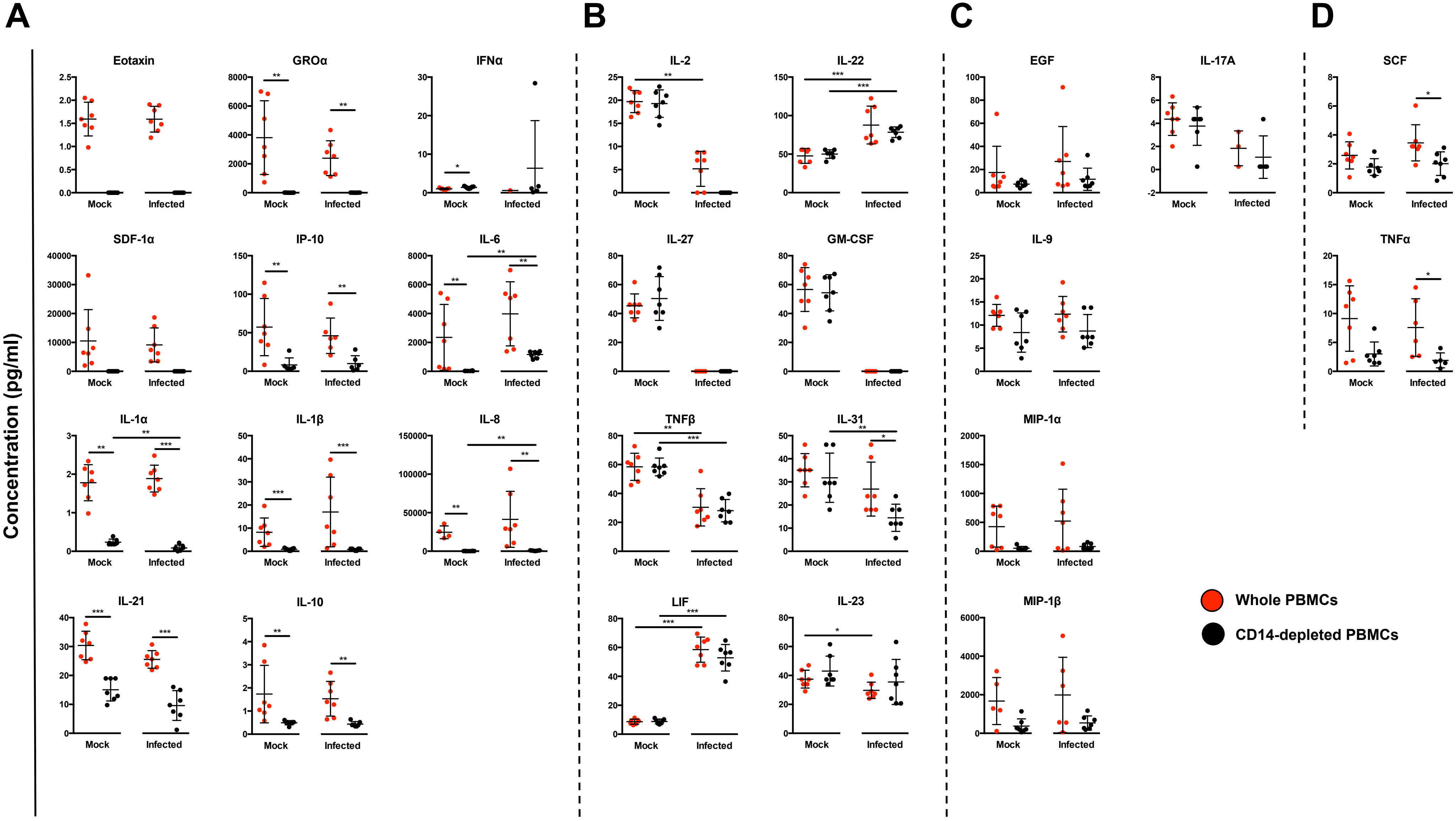

Supplementary Figure 4: Quantification of immune mediators. Immune mediators in the culture supernatant of ZIKV-infected PBMCs and CD14-depleted PBMCs were quantified using a 45-plex microbead-assay. Quantified immune mediators are grouped into four groups based on their profile: (**A**) mediators affected by depletion of CD14⁺ monocytes, (**B**) mediators affected by ZIKV infection, (**C**) mediators was not affected by both CD14⁺ monocytes depletion and ZIKV infection and (**D**) mediators affected by depletion of CD14⁺ monocytes only after ZIKV infection. Data displayed were derived from seven donors. All data are presented as mean ± SD. *P < 0.05, **P < 0.01, ***P < 0.001, by Mann Whitney U test, two tailed. Abbreviations: PBMC, peripheral blood mononuclear cell.

Supplementary Figure 4_Lum et al., 2018
